# High Temperature, pH, and Hypoxia Cause Oxidative Stress and Impair the Spermatic Performance of the Amazon Fish *Colossoma macropomum*

**DOI:** 10.3389/fphys.2020.00772

**Published:** 2020-07-08

**Authors:** Jonatas S. Castro, Susana Braz-Mota, Derek F. Campos, Samara S. Souza, Adalberto L. Val

**Affiliations:** ^1^ Aquaculture Graduate Program, Nilton Lins University, Manaus, Brazil; ^2^ Laboratory of Ecophysiology and Molecular Evolution, National Institute of Amazonian Research, Manaus, Brazil

**Keywords:** aquaculture, antioxidant enzymes, climate change, comet assay, fish breeding, motility, tropical fish

## Abstract

The control of abiotic parameters is fundamental for fish survival, growth and reproduction. These factors have a direct effect on sperm quality. Thus, this study evaluated the effect of different temperatures (29, 31, 33, and 35°C), pHs (4 and 8), and hypoxia (1 mgO_2_ L^−1^) on sperm motility of *Colossoma macropomum* (tambaqui). The results indicated a longer duration of sperm motility at 29°C (50.1 ± 2.70 s) that progressively decreased when exposed to 35°C (31.2 ± 1.31 s) and hypoxia at pH 4 (27.4 ± 1.42 s) and pH 8 (30.44 ± 1.66 s; *p* < 0.05), respectively. Sperm oxygen consumption increased in hypoxia at both pH (pH 4 = 61.22; pH 8 = 54.74 pmol s^−1^). There was an increase in the activity of glutathione-S-transferase (GST) and superoxide dismutase (SOD), as well as in lipid peroxidation levels (LPO) and DNA damage in sperm exposed to higher temperatures and hypoxia. The pH 4 and pH 8 under normoxia did not affect the quality of *C. macropomum* sperm. These results suggest that water warming and acidification, consequences of climate changes, significantly affect the reproduction of *C. macropomum*, reducing the quality of spermatozoids during fertilization.

## Introduction

Climate changes are universal and are causing unprecedented environmental and life damage. Among the changes are higher temperatures, increases in precipitation rates, extreme drought, and changes in the habitat of terrestrial and aquatic species, which can result in reallocation and adaptation to survival (Isaak et al., 2012). Freshwater species face particular challenges within this scenario, worsened by increased pollution and over-exploitation of commercial fisheries, leading to declining fish populations (Wenger et al., 2011), that is, elimination of sensitive species, through direct and indirect effects on the biology of these organisms (Jeppesen et al., 2010), as well as on sperm quality and viability (Jenkins et al., 2018). Thereby, in order to minimize the impacts caused by fishing and climate change in natural environments, aquaculture is becoming increasingly important for the maintenance of fish stocks and for animal protein production. Therefore, targeted efforts to assess how fish in confined environments will be affected by the climate change in terms of reproductive efficiency are needed.

Dissolved oxygen (DO), pH, and temperature are important factors affecting survival, growth, and reproduction of fish (Caldwell and Hinshaw, 1994). In the Amazon region, these factors are influenced by diurnal variations of DO, seasonal flooding, thermal stratification, aquatic plants, and anthropogenic effects, such as eutrophication and global warming (Richards et al., 2009). The control of these factors is pivotal for the reproductive success of fish that have external fertilization (Dong et al., 2011), since sperm activity is only triggered when it comes into contact with water (Alavi and Cosson, 2006). Thus, sperm motility is strongly influenced by the activation medium (Dadras et al., 2016).

Sperm motility is a key factor determining semen quality being directly related to fertilization success, and is potentially used as a biomarker of environmental quality (Gallego et al., 2018). Negative effect of hypoxia, temperature, and pH on the spermatic quality of *Porichthys notatus*, *Sparus aurata* (Lahnsteiner and Caberlotto, 2012), and *Genypterus blacodes* (Dumorné et al., 2018) have been documented. The duration of sperm motility of farmedfish species is approximately 1 min; for this reason, accurate assessments using rapid and sensitive methods are needed to increase the efficiency of artificial fertilization (Kime et al., 2001; Rurangwa et al., 2004).

The motility and fertilization capacity of sperm are dependent on energy stored in the form of ATP, which in turn is strongly influenced by the oxygen concentration (Bencic et al., 1999). However, molecular oxygen, although necessary, can induce the production of reactive oxygen species (ROS), causing oxidative stress. During periods of environmental stress to which sperm are subjected at the time of activation, several antioxidant enzymes act to neutralize ROS (Dadras et al., 2016). The antioxidant capacity of semen can vary among fish species (Slowinska et al., 2013) and the formation of ROS can cause different types of damage, in particular lipid peroxidation of membranes, due to the abundance of polyunsaturated fatty acids, and DNA damage, with consequent loss of motility and decreased fertilization (Aramli and Kalbassi, 2016).

The tambaqui, *Colossoma macropomum* (Cuvier, 1818), is a migratory tropical fish species native from the Amazon and Orinoco basins (Araujo-Lima and Goulding, 1998), belonging to family *Serrasalmidae* (Mirade, 2010). The spawning of tambaqui coincides with the beginning of the flood, occurring in October/November and the species presents annual and total spawning. Mature individuals of *C. macropomum* have high fecundity, releasing more than one million oocytes. The fertilization is external and the species does not show parental care (Araujo-Lima and Goulding, 1998; Vieira et al., 1999). At the beginning of the flood season, individuals start their upstream migration into whitewater rivers, and when the gonads are fully developed they spawn along the grassy leaves that are being inundated with rising water. After spawning, the fish enter the floodplain areas where they feed mainly on fruits (Goulding and Carvalho, 1982;Araujo-Lima and Goulding, 1998).

This species is of great interest for researchers and producers due to its acclimatization facility, rapid growth, and great social and economic importance for the central region of the Amazon (Mendonça et al., 2009). Because it is a tropical species, *C. macropomum* is expected to be especially vulnerable to climate change, specifically to warming, because it has a narrower thermal sensitivity range compared to subtropical and temperate species and tends to live closer to its thermal limits (Lapointe et al., 2018). Therefore, even small temperature increases can lead to declining performance of individuals and populations (Tewksbury et al., 2008).

Considering that homeostasis of the sperm of *C. macropomum* is extremely important for the aquaculture industry, understanding how environmental parameters dependent on climate change affect sperm quality is critical in improving the methods and manipulations required for successful *in vitro* fertilization. Thus, the present study aims to investigate *in vitro* effects of temperature, pH and hypoxia on motility and antioxidant responses of the sperm of the Amazonian fish *C. macropomum*.

## Materials and Methods

### Semen Collection

Males of *C. macropomum* (*n* = 3, 60.4 ± 0.36 cm, 4.13 ± 0.05 kg) were selected from 600 m^2^ nurseries at the Center for Technology, Training and Production in Aquaculture (CTTPA; Balbina, Presidente Figueiredo, Amazonas – 1°55′54.4"S; 59°24′39.1"W). The fish were randomly selected for a light abdominal massage and in cranium-caudal direction, and those who released semen were chosen. The fish were recovered from this initial handling in 1000 L tanks with oxygen 5.54 ± 0.22 mg L^−1^, temperature 29.2 ± 0.07°C, and pH 7.73 ± 0.28 for a period of 6 h. Subsequently, spermiation was induced by intraperitoneal injection of crude extract of carp pituitary hormone (EBHC). The procedure was performed by injecting two boluses of EBHC. The first bolus was 0.5 mg/kg EBHC, and after an interval of 12 h, a second bolus of 1.0 mg/kg EBHC was injected. Six hours after injection of second hormone bolus, the semen was collected in graduated Falcon tubes, avoiding contact with water, blood, feces, or urine, thus preventing sperm activation. From each animal, 4 ml of semen was collected and immediately diluted 1:10 (50 μl semen: extender 450 μl) in Beltsville Thawing Solution (BTS-MINITUB®) and refrigerated at 4°C to be transported to the laboratory Ecophysiology and Molecular Evolution (LEEM) of the Brazilian National Institute for Research of the Amazon (INPA). All procedures and experimental manipulations used in this study were performed according to the Brazilian Guidelines for Animal Care and were approved by the Ethics Committee for the use of Animals of INPA, protocol number 004/2018.

### Activation

For activation of sperm motility, the following treatments were tested: temperature (29, 31, 33, and 35°C), pH (4 and 8), and interaction between pH and hypoxia (1 mgO_2_·L^−1^) in controlled activator solution (distilled water-0 mOsm kg^−1^). The different temperatures were maintained using thermostats HOPAR model (Aquarium Heater H-606). The pHs 4 and 8 were achieved by adding HCl 1% and NaOH 1%, respectively. The pH values were then monitored using a digital pH meter (Ohaus, Starter 3100). Hypoxia was adjusted with the addition of gaseous nitrogen until the concentration of 1 mgO_2_ L^−1^. The sperm were activated in the proportion 2:20 (v:v), in a closed environment with controlled temperature (23°C). Immediately after activation, sperm were observed under an optical microscope (Leica DM500; 40x), by the same observer to avoid the subject bias of the analyzes. The motility time was monitored with the aid of a timer (s) being evaluated from the start of movement until 100% sperm immobility. To identify the percent of sperm motility, a scale from 0 to 100% was used, according to Cosson et al. (2008), where: 1 = 0–5%, 2 = 5–25%, 3 = 25–50%, 4 = 50–75%, 5 = 75–100%. For each treatment, nine replicates were performed in triplicate.

### Oxygen Consumption of Sperm

Oxygen consumption of sperm was monitored using a high-resolution respirometer (Oxygraph-2 K, Oroboros Instruments) equipped with a Peltier thermostat and an electromagnetic stirrer. The treatments (temperature, hypoxia, and pH) were controlled within two 2 ml closed Oxygraph chambers. With the aid of injection cannulas, 100 μl of semen were added, immediately activated inside the chambers. Oxygen consumption was recorded immediately after activation until cessation of sperm respiration. Recordings of new samples were performed at intervals of 5 min between exposures, to the complete stabilization of the equipment. Data are expressed as pmol·s^−1^·cm^−3^.

### Biochemical Analysis

For analysis of antioxidant enzymes, 200 μl of semen from each fish was activated, with five replicates in triplicate for each treatment and after motility cessation, it was immediately immersed in liquid nitrogen. The semen samples were homogenized in a buffer solution (Tris base 200 mM, ethylene diamine tetraacetic acid (EDTA) 1 mM, dichloro-diphenyl-trichlorethane (DDT) 1 mM, sucrose 500 mM, KCl 150 mM, and pH 7.6) and centrifuged at 9,000 *g* for 10 min at 4°C. The supernatant was used for analysis of the activity of glutathione S-transferase enzyme (GST), superoxide dismutase (SOD) and catalase (CAT), and for determination of lipid peroxidation (LPO) levels. GST activity was determined as described by Keen et al. (1976), considering absorbance changes at 340 nm using 1-chloro-2,4-dinitrobenzene (CDNB) as the substrate. GST activity was calculated as nmol of conjugate CDNB·min^−1^·mg^−1^ protein, using the molar extinction coefficient 9.6 mM·cm^−1^. SOD activity was quantified from the inhibition of the reduction rate of cytochrome c influenced by the xanthine/xanthine oxidase system at 550 nm (Flohé and Otting, 1984) and is expressed as U min^−1^·mg of protein^−1^. CAT activity was determined following the method of Beutler (1975) and is expressed as μmol H_2_O_2_·min^−1^·mg^−1^ protein. The inhibition rate of H_2_O_2_ decomposition was monitored by decreasing the absorbance at 240 nm. LPO levels were measured by the ferrous oxidation/xylenol orange (FOX) method as described by Jiang et al. (1991) which consists of the oxidation of Fe^+2^ to Fe^+3^ hydroperoxide in an acid medium and subsequent formation of Fe^3+^/xylenol orange in the presence of the stabilizer butylate hydroxytoluene (BHT). The formation of this complex was quantified by increased absorption at 560 nm and is expressed in μM cumene hydroperoxide (CHP)·mg^−1^ of sperm protein. Total sperm protein was determined spectrophotometrically at 595 nm, according to the colorimetric assay described by Bradford (1976) using bovine serum albumin (BSA) as standard. All assays were performed with a SpectraMax M2 spectrophotometer, Molecular Devices Inc., Sunnyvale, CA, USA.

### Genotoxic Damage

To verify the DNA integrity of *C. macropomum* sperm cells, unicellular gel electrophoresis (comet assay) was used, according to Cabrita et al. (2005). After experimental exposures, 5 μl of semen was added to 300 μl of RPMI. Previously, the experimental units were covered with 0.5% agarose and all procedures were completed in triplicate. After agarose drying, the slides were placed in the lysis solution (2.5 M NaCl, 100 mM EDTA, 10 mM Tris, pH 10–10.5, 1% Triton X-100, and 10% DMSO) for 90 min at 4°C. The slides were then submerged in alkaline sodium hydroxide and EDTA buffer (300 mM NaOH and 1 mM EDTA, pH > 13) for 20 min. The slides were electrophoresed for 20 min at 25 V, 300 mA, and 4°C. Subsequently, the slides were neutralized (0.4 M Tris, pH 7.5 at 4°C) and stained with silver nitrate solution (5% sodium carbonate, 0.1% ammonium nitrate, 0.1% silver, 0.25% tungstosilicic acid, and 0.15% formaldehyde) for 15 min at 37°C. A total of 100 spermatozoids were randomly quantified per slide, using an optical microscope (Leica DM205) with a magnification of 100x. Damage was classified according the size of the tail that represents the degree of fragmentation of the DNA. DNA fragments of damaged nucleus migrate when exposed to an electrical field (electrophoresis). Fragments of different sizes migrate at different speeds, forming the typical figure of a comet. Thus, during the electrophoretic run, the more intense the breaks in the DNA molecule, the smaller the fragments generated and the greater the extent of migration and so the comet tail (Singh et al., 1988; Collins et al. 2008). The cells were divided into five categories, according to tail size, ranging from 0 to 4, i.e., class 0 corresponds to intact DNA, without tail; class 1, low damage index; class 2, intermediate damage; class 3, high damage; and class 4, extreme damage.

### Statistical Analysis

Data are expressed as mean ± SEM. To compare the different treatments, an ANOVA one way, followed by Tukey’s *post-hoc* test when differences (*p* < 0.05) between means were detected. Data that did not meet the premises of linear models were log transformed. All analysis was performed using the R-3.5.1 software (R Core Team, 2018), with additional car package (Fox and Weisberg, 2011).

## Results

### Sperm Motility

**Figure 1:**
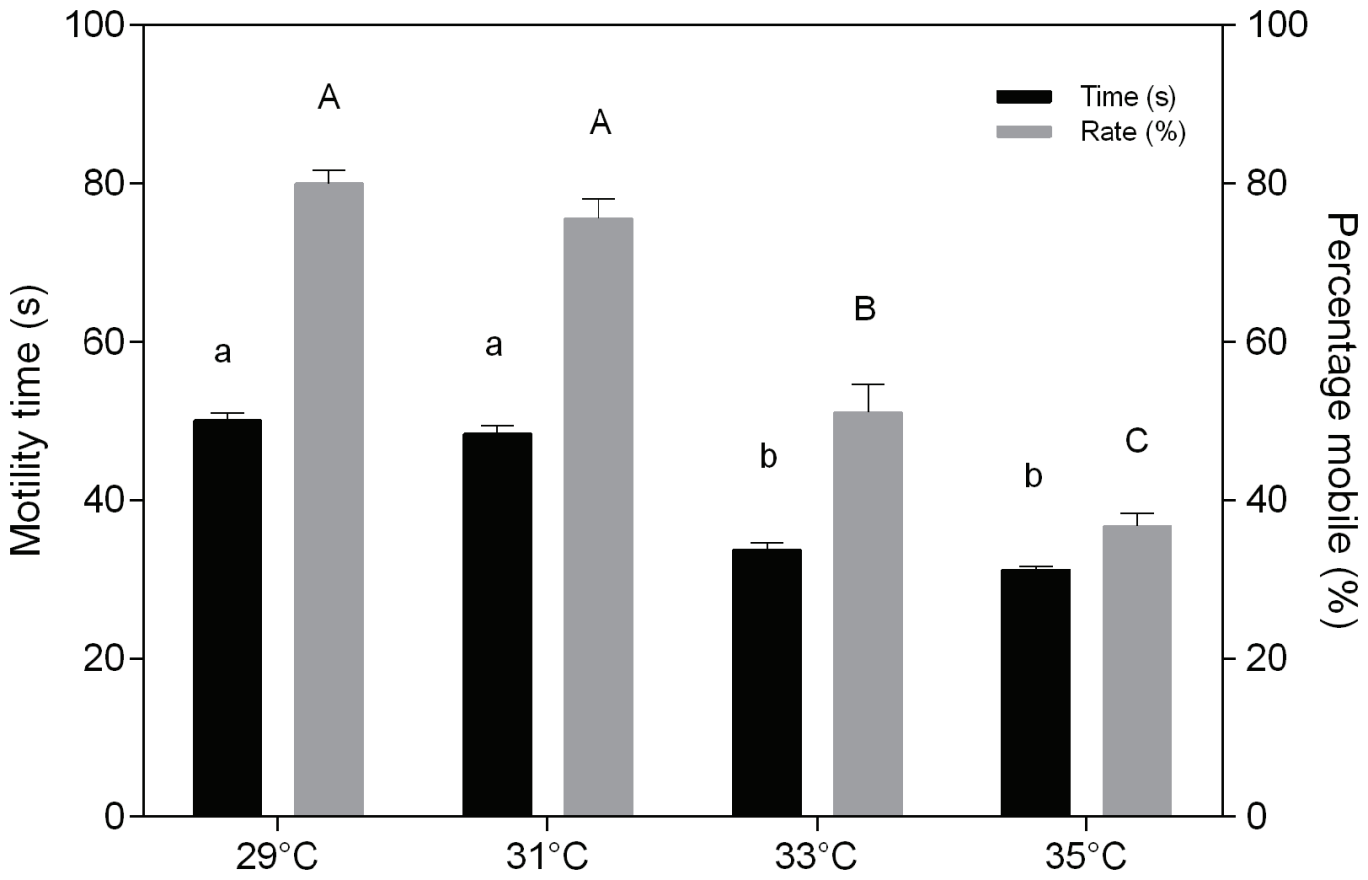
*In vitro* effect of temperature on time (s) and percentage mobile (%) of sperm of *Colossoma macropomum*. Data are presented as mean ± SEM. Lowercase letters indicate difference in motility time between treatments. Uppercase letters indicate differences in motility rate between treatments (*p* < 0.05).

**Figure 2:**
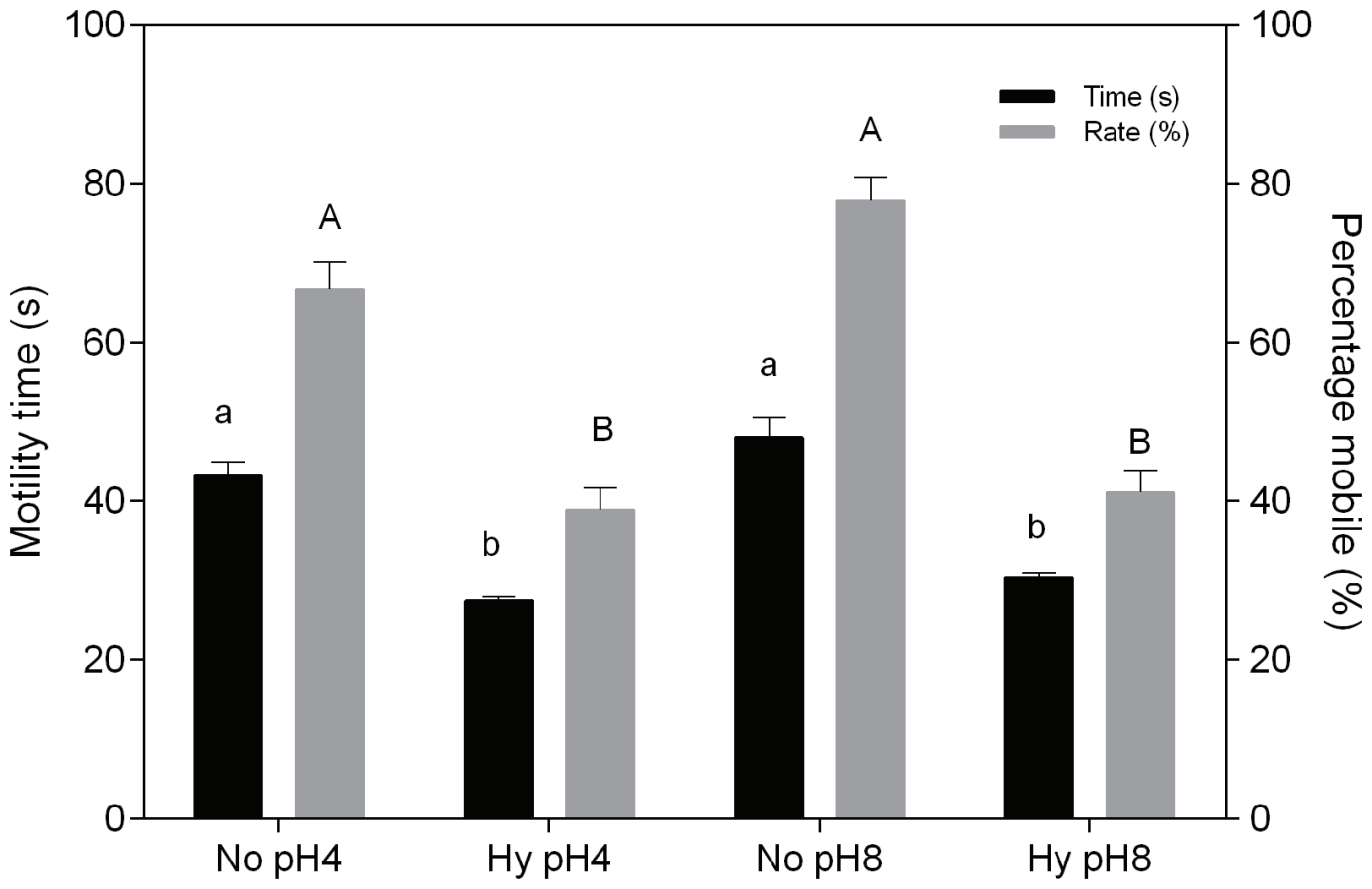
*In vitro* effect of pH and hypoxia (Hy) on time (s) and percentage mobile (%) of sperm of *C. macropomum*. Data are presented as mean ± SEM. Lowercase letters indicate differences in motility time between Hy and normoxia (No). Uppercase letters indicate differences in motility rate between Hy and No (*p* < 0.05).

The duration of *C. macropomum* sperm motility was 50.1 ± 2.7 s at 29°C and significantly reduced (*p* < 0.05) at 33°C (33.66 ± 2.58 s) and 35°C (31.22 ± 1.31 s; Figure 1). The percentage of mobile cells showed no differences at 29 and 31°C, but decreased significantly at 33 and 35°C (Figure 1). Exposure to pH 4 and pH 8 in hypoxia caused a significant reduction in motility time and percentage of mobile sperm (*p* < 0.05; Figure 2). Motility time and percentage of mobile sperm showed no differences when exposed to pH 4 and pH 8 in normoxia (*p* > 0.05; Figure 2).

### Oxygen Consumption of Sperm

**Figure 3:**
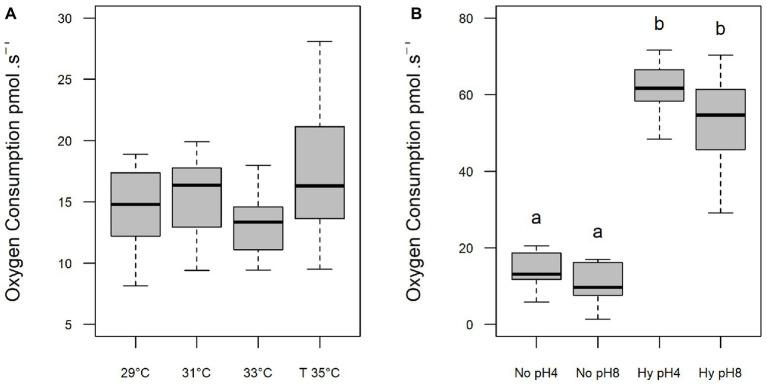
Oxygen consumption (pmol⋅s^−1^) of *C. macropomum* sperm activated at different *in vitro* temperatures **(A)** and at pH 4 and pH 8 in No (5.5 mgO_2_/L) and Hy (1 mgO_2_/L). **(B)** Lowercase letters indicate differences between treatments.

The oxygen consumption of sperm was not altered with increasing temperatures (*p* > 0.05; Figure 3A) or under different pHs within the same treatment (Figure 3B). However, exposure to hypoxia resulted in an increase in oxygen consumption at both pH 4 (61.22 pmol s^−1^) and pH 8 (54.74 pmol s^−1^) compared to normoxia at pH 4 (14.20 pmol s^−1^) and pH 8 (10.59 pmol s^−1^; *p* < 0.05; Figure 3B).

### Biochemical Analysis

**Figure 4:**
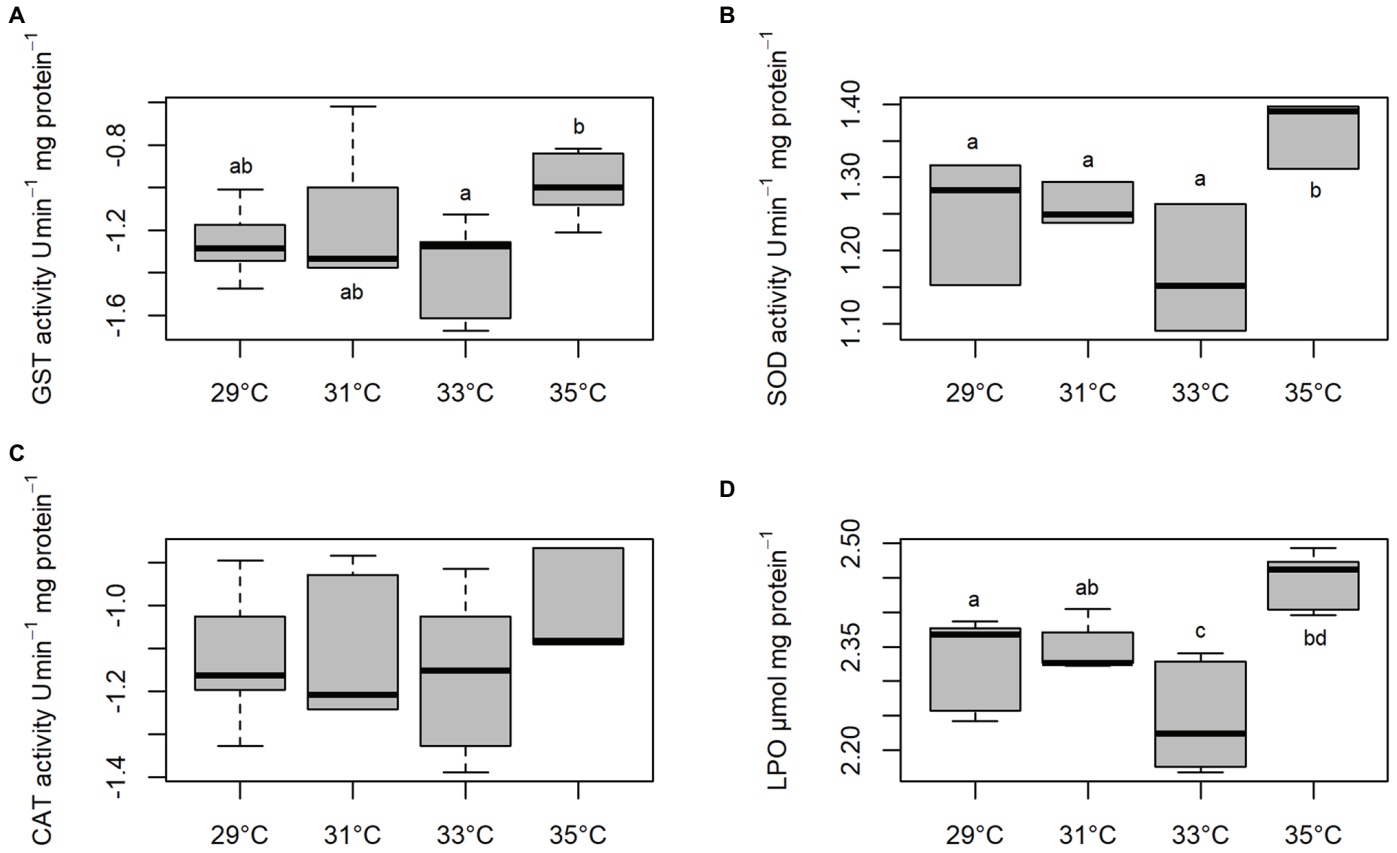
*In vitro* effect of temperature on Glutathione-S-transferase (GST), **(A)** superoxide dismutase (SOD), **(B)** catalase (CAT), **(C)** and lipoperoxidation (LPO) **(D)** levels in sperm of *C. macropomum*. Lowercase letters indicate differences between treatments.

**Figure 5:**
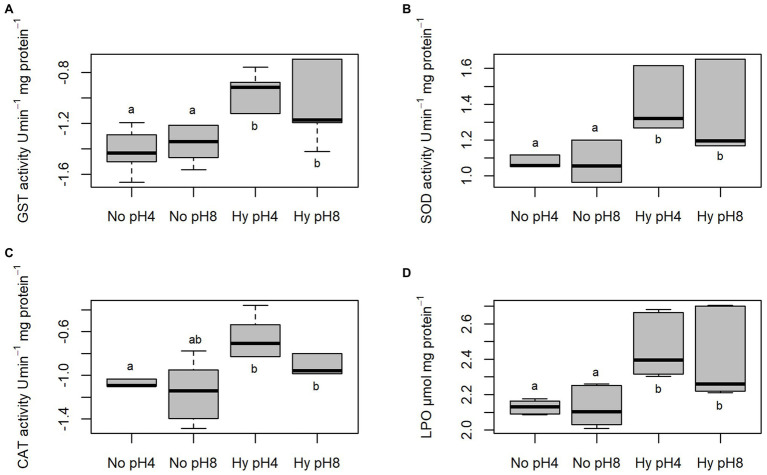
*In vitro* effect of pH in No (5.5 mgO_2_/L) and Hy (1 mgO_2_/L) on GST, **(A)** SOD, **(B)** CAT, and **(C)** LPO, **(D)** in sperm of *C. macropomum*. Lowercase letters indicate differences between treatments.

Activities of GST, SOD, and CAT and levels of LPO of *C. macropomum* sperm exposed to different temperatures are shown in Figure 4. GST activity was increased at 35°C, differing from sperm exposed to 33°C (*p* < 0.05), and similar to other treatments (*p* > 0.05; Figure 4A). SOD activity was significantly increased at 35°C compared to all other treatments (*p* < 0.05; Figure 4B). CAT did not differ in any of the treatments (*p* > 0.05; Figure 4C). Exposure to 35°C caused an increase in LPO levels compared to cells exposed to 29 and 33°C, while for sperm exposed to 33°C LPO levels were reduced compared to other groups (*p* < 0.05; Figure 4D). Low oxygen concentration exposure during sperm activation caused an increase of GST and SOD activities (Figures 5A,B). CAT activity at pH 8 in normoxia was equal to pH 4 normoxia (*p* > 0.05) and showed a similar not significant increase of sperm exposed to hypoxia at both pHs (*p* < 0.05; Figure 5C). Increased enzymatic activities in hypoxic environments contributed to increased LPO levels (Figure 5D). Normoxic environments at pHs 4 and 8 did not affect the activities of SOD and GST, as well as the LPO levels (*p* > 0.05; Figure 5).

### Genotoxic Damages

**Figure 6:**
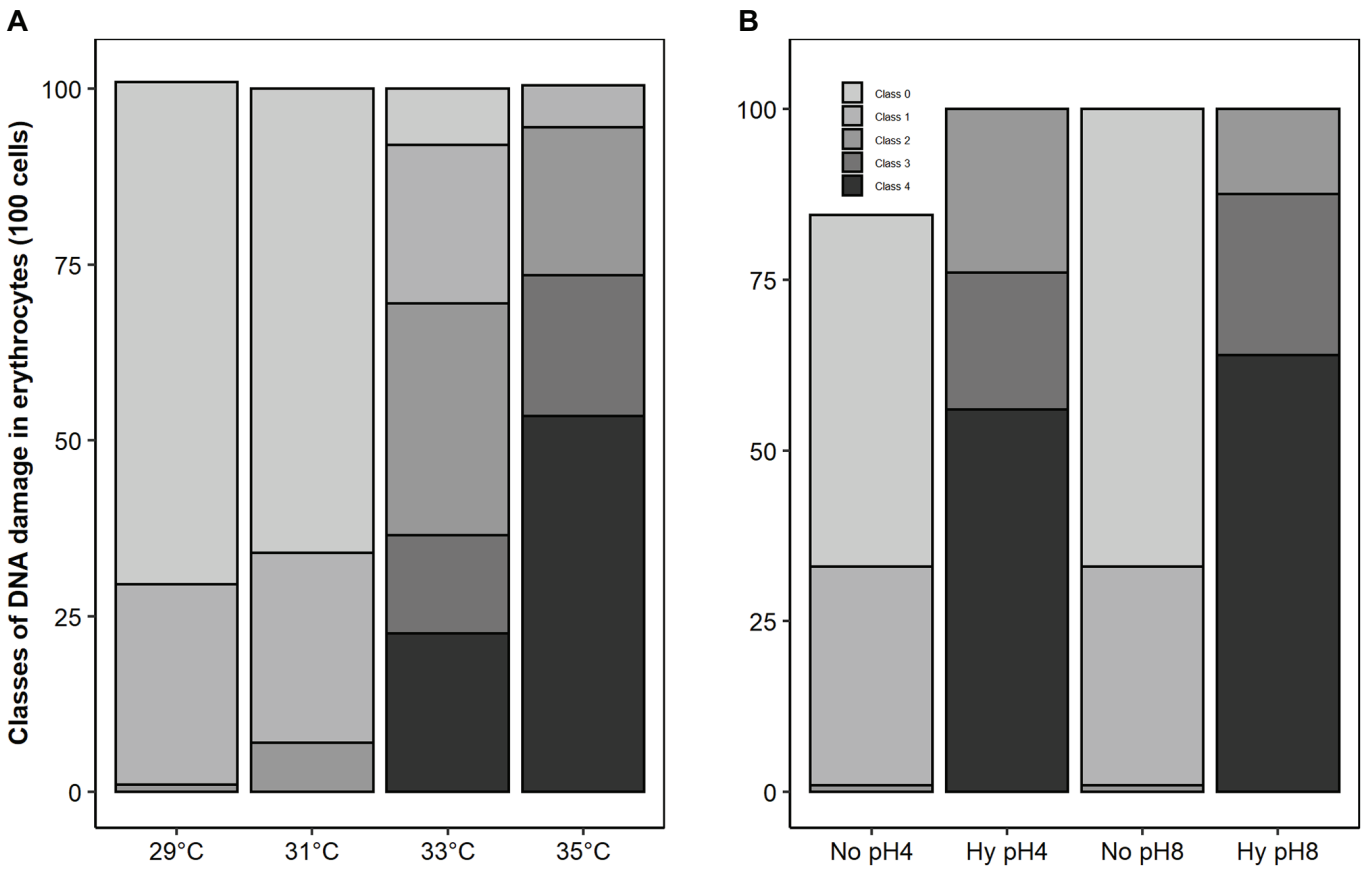
Frequency of DNA damage of sperm of *C. macropomum* exposed to different temperatures **(A)** and exposed to pH 4 and pH 8 in No and Hy **(B)** *in vitro*.

Genetic damage to sperm of *C. macropomum* increased at higher temperatures (33 and 35°C) compared to sperm activated at 29 and 31°C (Figure 6). At 29 and 31°C, there was a prevalence of intact DNA with about 71 and 66%, respectively. At the highest temperature, 35°C, approximately 60% of the sperm cells had extreme damage, class 4 (Figure 6). Activated spermatozoa under normoxia showed the highest rate of intact cells (pH 4 = 43%, pH 8 = 57%), but low oxygen activation medium on both pHs caused an increased classes 2, 3, and 4 damages, considered intermediate, high, and extreme, respectively (Figure 6).

## Discussion

### Temperature Effects

Sperm motility, a critical semen characteristic, depends on activation media (Williot et al., 2000) that strongly influence the fertilization in teleosts (Bera et al., 2016). In the present study, sperm exposure to increased temperature (33 and 35°C) caused a reduction on time and percentage of mobile sperm (Figure 1) without altering the oxygen consumption (Figure 3A). These findings corroborate the report of Lahnsteiner and Caberlotto (2012) for *S. aurata* and Dadras et al. (2016) for *Cyprinus carpio*, which showed that higher temperatures caused lower percentage of mobile sperm. According to Purchase et al. (2010), fish sperm has limited energetic resources; thus, temperature increase causes a motility decrease due to the acceleration of the metabolic processes. Low temperatures reduce the intensity of flagellar movement significantly, prolonging motile duration of spermatozoids (Alavi and Cosson, 2006). In the present study, the high temperature accelerated the swimming speed of *C. macropomum* sperm cells, increasing the metabolic consumption of the cells and decreasing the motility time and the percentage of mobile cells. Activated sperm at low temperatures consume metabolic energies more slowly, increasing the duration of these cells, corroborating the report of Alavi and Cosson (2006), who found that low temperatures reduce the intensity of flagellar movement, significantly prolonging the mobile duration of sperm.

As a result, at lower temperatures, the consumption of ATP is reduced, which is directly related to the longer motility time (Lahnsteiner and Caberlotto, 2012). Dzyuba et al. (2019), studying the tropical species *Oreochromis niloticus*, showed that higher temperatures induce an increase in sperm motility velocity, which is accompanied by a reduction in motility time. The reduction in time and percentage of mobile sperm at the higher temperatures observed in the present study may be related to increased energy demands, which was not compensated for, since oxygen consumption remained constant at all temperatures. Therefore, our results suggest that semen from tambaqui has limited energy supply to deal with increased temperature, suggesting high vulnerability of this species under warming scenarios.

Another factor that may explain the lower percentage of mobile sperm observed at the higher temperature is related to the oxidative damages caused by ROS production, as observed in the present study. The exposure of sperm cells at 35°C resulted in an increase in the activity of antioxidant enzymes (SOD and GST; Figure 4); however, such increase was not enough to avoid damage to macromolecules, since there was an increase in lipoperoxidation and DNA damage (Figures 4, 6A). Oxidative stress can induce metabolic disorders that reduce sperm motility. As suggested by Aramli and Kalbassi (2016), oxidative stress causes disturbances in the sperm metabolism, which results in decreased sperm motility.

The oxidative damage observed in the present study suggests a inefficiency in the neutralization of ROS by the antioxidant defenses, since even with the increase of antioxidant enzyme activity, such as SOD, sperm presented high levels of lipoperoxidation and DNA damage. Aramli and Kalbassi (2016) also showed that the increase of the antioxidant defenses in the species *Acipenser persicus* was accompanied by the increase of oxidative damages. Fish sperm have characteristics that make them susceptible to damage related to oxidative stress, considering the high amount of polyunsaturated fatty acids of sperm membrane (Shaliutina et al., 2013).

Studies have shown that temperature increases potentially induce oxidative stress in different fish tissues (Wiens et al., 2017), including sperm cells, as presented in this paper with the increase in antioxidant enzymes, contributing to the increase in lipid peroxidation levels, as shown by Dadras et al. (2016) when evaluating the sperm of *C. carpio*, identifying that the lipid peroxidation in these cells is temperature sensitive. Lipids are probably used as a source of energy by sperm to increase motility duration (Baeza et al., 2015) that plays an important role in fertilization capacity (Gholami et al., 2011). The lipid peroxidation of these cells is associated with deterioration of membrane function, decreased fluidity, and increased non-specific permeability to ions and other transmembrane transport processes (Wong-Ekkabut et al., 2007).

Although *C. macropomum* sperm cells were activated at the high temperature evaluated in this experiment, they showed a significant increase in tail damage (class 4). The evaluation of fish sperm DNA damage is important for fish farming, since the loss of genetic information or the appearance of larval deformities is detrimental to future generations (Cabrita et al., 2005; Lewis and Galloway, 2009). Due to the reduced motility time of freshwater fish spermatozoid, the activation environment is critical for the successful delivery of paternal DNA to the egg (Gazo et al., 2015). However, oxidative stress is highly detrimental to DNA integrity, since this damage is not limited to the direct effect on the fragmentation of chromatin; it can also alter gene expression or induce epigenetic deregulation (Chervona and Costa, 2012). The extreme DNA damage of *C. macropomum* sperm caused by activation at high temperatures is similar to the results found by Lacaze et al. (2011) in assessing DNA damage of the sperm of *Gammarus fossarum*. The authors explain that the increase in damage is associated with an overall stress caused by increased temperature and decreased oxygen concentration. Our results corroborate the findings of De Andrade et al. (2004), who found an increase in DNA damage of *Dreissena polymorpha* and *Netuma* sp. exposed to 37°C. Despite increased DNA damage in various species of fish exposed to various stressors, Pérez-Cerezales et al. (2010) reported that DNA damaged spermatozoids are capable of performing fertilization and that the oocyte can repair this damage to some extent in *Oncorhynchus mykiss*. These authors stated that when the DNA fragmentation rate is high, the oocyte repair capacity is insufficient, and infertility rate increases considerably.

### Effects of pH and Hypoxia

In the present study, activation media pHs (pH 4 and pH 8) did not influence the sperm quality of *C. macropomum*. This may be related to the adaptation of this species to large pH range that drives resilience of sperm cell to pH changes and so, possibly, increasing reproductive success. Aride et al. (2004) reported that in confined environments *C. macropomum* is able to tolerate large variations in pH, which was confirmed by Wood et al. (2018). Cosson (2004) showed that the pH of activating medium usually affects motility, but in small proportions. In addition, the external pH can reduce or prolong motility, but it is not the main environmental factor affecting initial motility (Cosson et al., 2008). The results found in this study are similar to the findings by PerchecPoupard et al. (1997), who showed reduced pH impact on carp sperm motility, indicating that fish that inhabit tropical/subtropical environments are possibly more resistant to pH variation. However, for species that inhabit temperate environments such as *Samotrutta*, variations in pH affect sperm motility (Dziewulska and Domagała, 2013).

Hypoxia exposure affected sperm quality of *C. macropomum*, suggesting that hypoxia, a common environmental condition in tropical waters and aquaculture, is highly detrimental to the reproduction of this tropical species. The results observed in the present study are in agreement with Wu et al. (2003), which suggested that hypoxia caused reduction of sperm motility in carp and other reproductive dysfunctions, such as the retardation of gametogenesis. Thomas et al. (2007) evaluated the formation of sperm cells of *Micropogonias undulates* and found that sperm production, gametogenesis and all stages of spermatogenesis were impaired in hypoxic environments.

The decrease in the time and percentage of mobile sperm of *C. macropomum* under hypoxic conditions was accompanied by an increase in oxygen consumption (Figure 3B). At low oxygen, sperm cells present an increase in the rate of flagellar motility, not measured in the present study, which resulted in decreased motility time due to the rapid consumption of ATP. As a result, oxygen consumption increased. Fitzpatrick et al. (2009) showed that sperm velocity of *P. notatus* exposed to hypoxia was positively related to oxygen consumption. Both species breed in hypoxic environments. During the breeding period, males of *P. notatus* are trapped in small pools facing drastic hypoxia, similar to *C. macropomum* in their natural environments. It is known that the mitochondria of fish sperm supply ATP demand aerobically (Dzyuba et al., 2017). According to Cosson (2010), as the ATP reserve is depleted along the spermatozoid activity, there is a gradual decrease of the flagellum movement, settling off the movement of the cell. In aquaculture, fish are commonly kept under artificial conditions, exposed to various adverse conditions, which may contribute to the establishment of hypoxic environments (Rurangwa et al., 2004). Therefore, sperm exposed in these environments consume more quickly ATP reserves, directly affecting the duration of motility and the efficiency of fertilization. In addition, recent studies suggest that changes in sperm quality may be related to changes at the molecular level, such as changes in gene expression or epigenetic factors. Wang et al. (2016) verified the occurrence of epigenetic changes in the sperm methyloma of *Oryzias melastigma* exposed to hypoxia, as well as differential expression of genes and proteins involved in spermatogenesis and gene silencing, which was associated with a reduction in sperm motility, even in generations that never had been exposed to hypoxic conditions.

We also observed that exposure to hypoxia caused an increase in antioxidant activity (SOD, CAT, and GST; Figure 5), which suggests an increased capacity to prevent ROS effects. However, despite the higher activity of antioxidant enzymes under hypoxia condition, there was an increase in lipoperoxidation levels and DNA damage. Thus, the oxidative damages observed in the present study may be related to loss of sperm quality under hypoxic conditions. It has been shown that hypoxia induces oxidative stress in different fish tissues (Mustafa et al., 2011). The results of our study are similar to those of Bera et al. (2016), evaluating the activity of antioxidant enzymes in the testes and ovary of *C. carpio* in hypoxic environments. The authors reported an increase in CAT and SOD activities compared to tissues under normoxia. Other authors report the relationship between loss of sperm quality and oxidative damage in fish sperm under different stress conditions (Dadras et al., 2016; Wiens et al., 2017). Shaliutina et al. (2013) also showed a relationship between loss of sperm quality and oxidative stress in *Acipenser gueldenstaedtii* and *Acipenser bae*. Taken together, these results suggest that tambaqui reproduction is especially sensitive to disturbances caused by exposure to hypoxia.

## Conclusions

The elevation of temperature and the decrease in DO, factors associated among others to climate change, will negatively affect sperm quality of *C. macropomum*. No pH effects on spermatozoids of *C. macropomum* were observed. This study provides basic information for future research on sperm quality of tropical fish in confined environments. However, further investigations are needed to describe the mechanisms involved in metabolism and sperm motility in *C. macropomum* under warm hypoxic environments. In general, the present results suggest that *C. macropomum* sperm are vulnerable to warm hypoxic waters, conditions associated with global warming, and may impair the reproduction of the species.

## Data Availability Statement

The datasets generated for this study are available on request to the corresponding author.

## Ethics Statement

The animal study was reviewed and approved by Brazilian Guidelines for Animal Care and were approved by the Ethics Committee for the use of Animals of INPA, protocol number 004/2018.

## Author Contributions

AV and JC designed the work. JC performed the experiment, collected data, analyzed and drafted the original version of the manuscript. SS, SB-M, and DC helped with laboratory analyzes, discussed and reviewed the manuscript and AV supervised this work, obtained financial support, discussed and revised the manuscript. All authors contributed to the article and approved the submitted version.

## Conflict of Interest

The authors declare that the research was conducted in the absence of any commercial or financial relationships that could be construed as a potential conflict of interest.

## Abbreviations

ADAPTA, Adaptations of aquatic biota of the Amazon; ATP, Adenosine triphosphate; BHT, Butylate hydroxytoluene; BSA, Bovine serum albumin; BTS, Beltsville thawing solution; CAPES, Coordination for the improvement of higher education personnel; CDNB, 1-Chloro-2,4-dinitrobenzene; CHP, Cumene hydroperoxide; CNPq, Brazilian National Research Council; CTTPA, Center for Technology Training and Production in Aquaculture; DDT, Dichloro-diphenyl-trichlorethane; DMSO, Dimethyl sulfoxide; EBHC, Crude extract of carp pituitary hormone; EDTA, Ethylene diamine tetra-acetic acid; ROS, Reactive oxygen species; FAPEAM, Amazonas State Research Foundation; FOX, Ferrous oxidation/xylenol orange; GST, Glutathione S transferase; HCL, Hydrochloric acid; INPA, National Institute for Research of the Amazon; LEEM, Laboratory of Ecophysiology and Molecular Evolution; LPO, Lipoperoxidation; NaOH, Sodium hydroxide; SEM, Standard error of mean; SOD, Superoxide Dismutase.
